# The usefulness of a pectoralis major myocutaneous flap in preventing salivary fistulae after salvage total laryngectomy

**DOI:** 10.1590/S1808-86942012000400019

**Published:** 2015-10-20

**Authors:** Alexandre Andrade Sousa, Sebastião Maurício de Oliveira Castro, José Maria Porcaro-Salles, João Marcos Arantes Soares, Gustavo Meyer de Moraes, Jomar Rezende Carvalho, Guilherme Souza Silva

**Affiliations:** 1 MSc in Surgery at FM/UFMG, Member of the SBCCP (Professor in the Department of Surgery at FM/UFMG, Member of the Head and Neck Surgery Group at the Alfa Institute of Gastroenterology at HC/UFMG, Coordinator of the HNS Group - Baleia Hospital); 2 MD, General Surgeon (Medical Resident on Head and Neck Surgery); 3 MSc in Surgery at the Medical School of UFMG, Member of the Brazilian Association of Head and Neck Surgeons (Professor in the Department of Surgery at the Medical School of UFMG, Coordinator of the Head and Neck Surgery Group at the Alfa Institute of Gastroenterology of the UFMG University Hospital); 4 PhD in Surgery at FM/UFMG, Member of the SBCCP (Professor in the Department of Surgery at FM/UFSJ); 5 MSc in Surgery at FM/UFMG (Member of the Head and Neck Surgery Group at the Alfa Institute of Gastroenterology at HC/UFMG); 6 MSc, Member of the SBCCP (Member of the Head and Neck Surgery Group at the Alfa Institute of Gastroenterology at HC/UFMG); 7 Head and Neck Surgeon (Member of the Head and Neck Surgery Group at the Alfa Institute of Gastroenterology at HC/UFMG)

**Keywords:** digestive system fistula, laryngectomy, postoperative complications, surgical flaps

## Abstract

Salvage laryngectomy in patients treated with organ preservation protocols is associated with high rates of postoperative complications. The use of non-irradiated tissue flaps in pharyngeal reconstruction could reduce the incidence of these complications.

**Objective**: This study aims to evaluate the usefulness of the pectoralis major myocutaneous flap in preventing salivary fistulae during the postoperative period of salvage total laryngectomy (TL).

**Materials and Method**: This retrospective study enrolled 31 patients operated between April of 2006 and May of 2011. All patients had advanced cancer at the time of the salvage procedure and had been treated with chemoradiotherapy or radiotherapy alone. Pharyngeal reconstruction was performed using pectoralis major myocutaneous flap in 19 cases (61%); primary wound closure occurred in 12 patients (39%).

**Results**: Salivary fistulae occurred in 16% of the patients who received the flap and in 58% of the patients with primary closure of the pharynx (*p* < 0.02). No statistically significant differences were noted between the groups with respect to the mean time for fistula formation, reintroduction of an oral diet, or use of a nasoenteric tube for feeding.

**Conclusion**: The pectoralis major myocutaneous flap was found to reduce the incidence of salivary fistulae in salvage laryngectomy procedures.

## INTRODUCTION

The treatment of head and neck squamous cell carcinoma (SCC) has changed substantially in the last few years^1^. The development of organ preservation protocols and primary chemoradiotherapy (CRT) for a subgroup of patients with advanced laryngeal cancer resulted in organ preservation rates of 64% and global survival similar to the levels seen in patients offered laryngectomy followed by radiotherapy, a treatment then considered the standard for this type of cancer^2^, ^3^, ^4^.

Despite the efficacy of chemoradiotherapy, a considerable number of patients still require salvage surgery - usually in the form of total laryngectomy - when the disease persists or recurs^5^, ^6^. However, a number of studies have shown high complication rates associated with salvage laryngectomy due to the effects of radiotherapy combined with chemotherapy upon wound healing^3^, ^7^. More specifically, pharyngocutaneous fistulas - the most common post-laryngectomy complication - occur more frequently in this population and may affect more than 50% of the patients^8^. This complication leads to increased morbidity, prolonged hospitalization, and greater hospital care expenditure^9^, ^10^. The same happens to patients with early recurring laryngeal tumors after radiotherapy alone^5^, ^6^.

In an attempt to prevent or reduce the incidence of salivary fistulas, some authors have advocated the routine use of non-irradiated tissue to reinforce and close the larynx. Candidate tissues range from pectoralis major myocutaneous flaps, sternocleidomastoid muscle collar flap, to microvascular flaps^2^.

This study aims to assess the use of pectoralis major myocutaneous flaps to close the pharynx in patients submitted to therapy with chemotherapy and/or radiotherapy followed by total laryngectomy (TL) or salvage total pharyngolaryngectomy (TPL) and compare the incidence of salivary fistulas in flap and primary pharyngeal closure patients.

## MATERIALS AND METHODS

This retrospective study included the charts of patients diagnosed with relapsing squamous cell carcinoma (SCC) after primary treatment with radiotherapy alone or radiotherapy combined with chemotherapy seen from April of 2006 to May of 2011. All patients underwent TL or salvage TPL followed by primary pharyngeal closure or placement of a pectoralis major myocutaneous flap to repair the pharyngeal tract. The choice of using the flap or not was not randomized and was based on the intraoperative preference and judgment of the surgeon.

This study was approved by the Ethics Committee on April 5, 2010 and granted permit # 0584.1.203.000-09.

The following parameters were compared: primary pharyngeal closure and repair with pectoralis major myocutaneous flap, index and mean time until the appearance of a salivary fistula, use of nasoenteral feeding tube, time until reintroduction of oral feeding. Previous tracheostomy was also investigated as a possible predisposing factor for the formation of salivary fistulas.

Only patients with primary tumors located in the larynx or hypopharynx, with failed treatment after chemotherapy and/or primary radiotherapy, and submitted to TL or TPL were included in the study. Patients submitted to partial laryngectomy, partial base of tongue resection were excluded.

Statistical analysis was done with software package SPSS release 13.0; all tests considered a level of statistical significance of 5%.

## RESULTS

Thirty-one patients with relapsing or residual SCC in the larynx or hypopharynx were operated. The diagnosis was confirmed through pathology tests.

Patient mean age was 65.5 years (43-84 years). Data on gender, nutritional status, and previous tracheostomy can be seen on Table 1.

**Table 1:** Patient clinical characteristics (n = 31).

Variables		n	%
Gender	Male	30	97.0
	Female	1	3.0
Previous tracheostomy	Yes	17	55.0
	No	14	45.0
Nutritional status	Eutrophic	8	25.0
	Mild malnutrition	6	19.0
	Moderate malnutrition	1	3.0
	Not informed	22	31.0

The most frequent comorbidity was high blood pressure, seen in 19% of the patients. Other comorbidities were: chronic liver and coronary disease 6%, heart disease 3%. Sixty-six percent (22/31) of the patients had incomplete data on their associated comorbidities.

Primary tumor locations can be seen on Table 2.

**Table 2:** Primary tumor site (n = 31).

Tumor site	n	%
Supraglottis	2	6.0
Glottis	13	43.0
Subglottis	1	3.0
Transglottis	8	25.0
Hypopharynx	7	23.0

Patient staging according to the TNM classification of malignant tumors can be seen on Table 3.

**Table 3:** Patient TNM^11^ staging (n = 31).

Staging		n	%
T	T3	10	31%
	T4	22	69%
N	N0	24	75%
	N1	5	15%
	N2	1	3%
	N3	2	6%

Twenty-three percent of the patients (7/31) underwent pharyngolaryngectomy and 77% (24/31) were submitted to total laryngectomy. The neck was surgically approached in 33% of the patients the following way: radical bilateral neck clearance in 3% of the cases, unilateral radical neck clearance in 3% of the patients, bilateral jugular clearance in 18% of the cases, radical clearance on one side and jugular clearance on the other side in 3%, selective clearance of levels II-III in 3%, and in 6% of the patients there was no information on their charts. No type of clearance was performed in 61% of the patients (Table 4).

**Table 4:** Treatment offered.

	Variables	n	%
Type of surgery	Total laryngectomy	24	77.0
	Total pharyngolaryngectomy	7	23.0
Pharyngeal reconstruction	Pectoralis flap	19	61.0
	Primary closure	12	39.0
Neck clearance	Radical bilateral	1	3.0
	Radical unilateral	1	3.0
	Jugular bilateral	6	18.0
	Radical + jugular	1	3.0
	Superselective	1	3.0
	None	19	61.0

Pharyngeal reconstruction was performed with pectoralis major myocutaneous flap in 61% (19/31) of the cases, while primary pharyngeal closure was done in 39% (12/31) of the patients.

In terms of postoperative complications, the overall incidence of salivary fistulas was 32% (10/31). Fifty-eight percent (7/12) of the patients submitted to primary pharyngeal closure had salivary fistulas. Sixteen percent (3/19) of the myocutaneous flap patients had pharyngocutaneous fistulas (*p* < 0.02). There was no difference on the incidence rates of fistulas when the following variables were compared: T and N staging; tumor location. There was a trend for lower incidence rates among eutrophic patients when compared to patients with some degree of malnutrition (*p* = 0.09) (Tables 5 and 6).

**Table 5:** Salivary fistula incidence: comparison between pectoralis major flap and primary closure.

Variables		Pectoralis major flap	Primary closure	Total	*p*
Salivary	^Yes^	3	7	10	0.02
fistula	No	16	5	21	
Total		19	12	31

**Table 6:** Comparison between types of pharyngeal closure in relation to time of use of NET, reintroduction of oral feeding, and appearance of salivary fistula (n = 31).

Variables	Pectoralis major flap	Primaryclosure	^*p*^
Mean time on NET*	24.6	23.7	0.47
Mean time until reintroduction of oral feeding	19.3	14.4	0.8
Mean time to fistula appearance	12.6	9.5	0.47

* NET = nasoenteral tube.

Five in 17 (29%) patients submitted to previous tracheostomy and salvage laryngectomy had salivary fistulas, while four in 12 (33%) of the patients without previous tracheostomy had salivary fistulas (*p* = 0.56).

On the donor site, the surgical wound was affected by infection in 18% of the cases and hematoma in 10% of the patients. None of the patients had chyle fistulas, one patient had pneumonia and another died 35 days after surgery of pulmonary sepsis.

Likewise, when patients submitted to primary laryngeal closure and pectoralis major flap are compared, the mean time in days until the appearance of the fistula, the mean time of use of a nasoenteral tube, and mean time until the reintroduction of oral feeding were 9.5 and 12.6 days (*p* = 0.47), 23.7 and 24.6 days (*p* = 0.47) and 14.4 and 19.3 days (*p* = 0.80) respectively.

## DISCUSSION

The development of organ preservation protocols with chemoradiotherapy has changed the treatment given to patients with advanced laryngeal cancer. Aside from good local and regional control and survival rates similar to those seen in laryngectomy followed by radiotherapy, this combination of therapeutical approaches has the additional benefit of sparing the organ, allowing many patients to maintain good voice quality and little or no dysphagia. Chemoradiotherapy programs have thus become the standard of care for the eligible patient subgroups^1^, ^2^, ^5^, ^12^.

One of the consequences of this approach is the change on the role surgery plays in treating these patients. Surgery is no longer the primary therapy in these cases, and is being gradually reserved to treat cases of residual or relapsing tumors, when no other options are available^1^, ^2^, ^5^. Total laryngectomy (TL) and total pharyngolaryngectomy (TPL) are the standard salvage procedures after chemoradiotherapy, while partial laryngectomy is reserved for selected cases^6^.

Despite the benefits of conservative treatments, salvage laryngectomy in patients treated with chemoradiotherapy is associated with high postoperative complication rates^13^. Pharyngocutaneous fistulas are the most common complication, and usually occur within 7-10 days after surgery^9^. The incidence rates of salivary fistulas in this study was 32%, as also observed by other authors such as Varghese et al.^14^ - 41% and Putten et al.^6^ - 30%.

This complication may delay rehabilitation, adjuvant therapy^15^, and the reintroduction of oral feeding^10^, aside from increasing the length of hospitalization and care costs. Tsou et al.^16^ showed that the length of hospitalization of patients with pharyngocutaneous fistulas was 37.6 days, against 20.7 days for patients without fistulas.

Multiple risk factors for the formation of salivary fistulas have been described. Among them are T staging, associated comorbidities, neck clearance, pre and postoperative low levels of hemoglobin, low serum albumin, previous tracheostomy, type of closure, surgery time, and preoperative chemotherapy and radiotherapy^5^, ^7^, ^10^, ^15^. In this study, most of these variables were not related to increased incidence rates of postoperative salivary fistulas. On a meta-analysis, Paydarfar & Birkmeyer^15^ showed that preoperative radiotherapy increases the risk of pharyngocutaneous fistulas (RR, 2.28; ***p*** < 0.001), confirming the findings of other papers, in which fistula incidence rates ere higher in previously irradiated patients when compared to non-irradiated subjects as described by Ganly et al.^12^ (32% versus 12%, ***p*** = 0.012 respectively). The effect of radiotherapy translates into injuries to tissue microvasculature, resulting in myointimal fibrosis, endarteritis, and worsening of atherosclerosis, leading to a hypovascular, hypocellular, and hypoxic environment, reducing healing capacity, increasing the risk for fistula formation, its severity, and duration^5^, ^15^, ^17^.

The use of non-irradiated flaps has been recommended to reduce the incidence rates and the morbidities associated with salivary fistulas in salvage laryngectomy patients, even when thee is enough mucosa for a primary closure procedure^3^, ^7^. The rationale of this strategy revolves around using healthy tissue with abundant vascularization to maximize surgical wound healing, thus preventing local complications. Flaps have been used routinely in salvage laryngectomy procedures performed at head and neck surgery centers all over the world^8^.

Pectoralis major myocutaneous flaps have played an important role in head and neck reconstruction surgery since they were described by Ariyan in 1979. These flaps can be quickly fashioned, are viable and reliable, versatile in reconstruction procedures, located close to the site of resection, and allow the reconstruction to be done in one single procedure with low complication rates on the donor site^18^, ^19^, ^20^. When used in salvage laryngectomy, the myocutaneous flap may reduce salivary fistula incidence rates from 24% to 0% (*p* < 0.015), as shown by Patel & Keni^8^, and from 51% to 20% (*p* < 0.028) according to Righini et al.^21^. In our study, the incidence of salivary fistulas in myocutaneous flap patients was 16%, while primary laryngeal closure patients had a salivary fistula incidence rate of 58%. Additionally, pectoralis major myocutaneous flaps seem to limit fistula severity and reduce the need for more surgery in patients with salivary fistulas^2^, ^7^, although our study lacks the statistics to support this statement.

Similar results were achieved by Tsou et al.^16^, with reduced incidence rates of fistula in salvage pharyngolaryngectomy patients using radial forearm fasciocutaneous flaps.

As it is a regional flap, it does not require specific materials or teams specialized in microsurgical anastomoses; and it introduces low donor site morbidity^2^, ^21^.

Myocutaneous flaps could also reduce the risk of late complications such as stenosis, dysphagia, and use of feeding tubes as shown by Patel & Keni^8^; these findings were not observed in our study.

## CONCLUSION

Pectoralis major myocutaneous flaps in the reconstruction of the pharynx of pharyngolaryngectomy or salvage total laryngectomy patients after chemo and radiotherapy may significantly reduce the incidence of salivary fistulas.
